# The impact of multi-drug resistant *Pseudomonas aeruginosa* infections on acute pancreatitis patients

**DOI:** 10.1186/s12879-023-08230-y

**Published:** 2023-05-22

**Authors:** Di Wu, Wenjun Lu, Yilin Huang, Ge Qin, Huanmiao liu, Jie Xiao, Jie Peng

**Affiliations:** 1 Department of Gastroenterology, Xiangya Hospital, Central South University, Xiangya Road, Changsha, 410008 Changsha China; 2 Department of Emergency, Third Xiangya Hospital, Central South University, Changsha, China

**Keywords:** Acute pancreatitis, Multi-drug resistant Pseudomonas aeruginosa, Infection, Drug resistance, Risk factor

## Abstract

**Introduction:**

Acute pancreatitis (AP) accounts for a high proportion of digestive diseases worldwide and has a high risk of infection. *Pseudomonas aeruginosa*, a common pathogen of hospital infections, has been observed to increase the resistance rate to several antibiotics, causing difficulties in treatments. Our study aims to investigate the impact of the multi-drug resistant *Pseudomonas aeruginosa* (MDR-PA) infections on AP patients.

**Methods:**

At two Chinese tertiary referral centers for AP patients infected with MDR-PA, a retrospective case-control study with a 1:2 case-control ratio was performed. Comparisons were preformed between with/without MDR-PA infections and different drug-resistance of MDR-PA infections patients, respectively. Independent risk factors of overall mortality were assessed via univariate and multivariate binary logistic regression analyses, and the distribution and antibiotic resistant rates of strains were described.

**Results:**

Mortality in AP patients with MDR-PA infections was significantly higher than in those without MDR-PA infections (7 (30.4%) vs. 4 (8.7%), *P* = 0.048). The rate of prophylactic use of carbapenem for 3 days (0 vs. 50%, *P* = 0.019) and the incidence rate of multiple organ failure (MOF) (0 vs. 57.1%, *P* = 0.018) were remarkably higher in the carbapenem-resistant *Pseudomonas aeruginosa* group compared with the carbapenem-sensitive *Pseudomonas aeruginosa* group. In the multivariate analysis, the severe categories of AP (OR = 13.624, 95% CIs = 1.567–118.491, *P* = 0.018) and MDR-PA infections (OR = 4.788, 95% CIs = 1.107–20.709, *P* = 0.036) were independent risk factors for mortality. The resistance rates of MDR-PA strains were low for amikacin (7.4%), tobramycin (3.7%), and gentamicin (18.5%). The resistance rates of MDR-PA strains to imipenem and meropenem were up to, 51.9% and 55.6%, respectively.

**Conclusion:**

In AP patients, severe categories of AP and MDR-PA infections were both independent risk factors for mortality. Inappropriate use of carbapenem antibiotics and MOF were related to carbapenem-resistant *Pseudomonas aeruginosa* infections. Amikacin, tobramycin, and gentamicin are recommended for the treatment of AP patients with MDR-PA infections.

## Introduction

Acute pancreatitis (AP) is an inflammatory disease of the exocrine pancreas involving severe abdominal pain and multiple organ dysfunction, which may lead to pancreatic necrosis and persistent organ failure with high mortality [1–3]. Early immune overreaction in severe acute pancreatitis (SAP) patients, followed by immunosuppression, leads to secondary bacterial infections, persistent organ failure and other serious complications [4, 5]. When infections, especially multidrug-resistant (MDR) bacterial infections, occur in AP patients, they can accelerate sepsis, which in turn will have a great impact on the prognosis of the disease [6]. Previous studies have reported that infections caused by MDR bacteria are increasing in AP patients due to prolonged hospitalization and intensive care unit (ICU) stays [7, 8].

*P. aeruginosa* infections are frequently correlated with increased rates of morbidity and mortality in severely ill patients [9]. Critically ill and immunocompromised patients are susceptible to life-threatening infections caused by *P. aeruginosa*, a frequent bacterium of nosocomial infection, with increasing incidences [10–12]. In the ICU, the incidence rate of *P. aeruginosa* is 14.5%, of which 48.7% are MDR, with an increase in resistance to carbapenems and polymyxin [13]. *P. aeruginosa* infections may compromise the selection of antimicrobial therapy because of MDR and the complexity of the patients affected by these serious infections [14]. Carbapenem antibiotics have often been used, even overused, in AP patients complicated with infections due to their good tissue penetration for the pancreas and excellent anaerobic coverage. However, this may induce carbapenem-resistant *Pseudomonas aeruginosa* (CRPA) infections [15]. Furthermore, the overuse of antibiotics which can lead to an increase in CRPA, has been mention in the World Health Organization classifing the CRPA as a ‘critical priority’ [16, 17].

However, the time point for antibiotics use in the treatments of MDR bacterial infections in AP patients is controversial [18]. There are currently no reports on AP patients complicated with MDR-PA infections, implying that significant gaps remain in this field. The objectives of our study are to: (1) evaluate the effects of MDR-PA infections on AP patients; (2) investigate the differences between high- and low-level drug-resistance of MDR-PA infections; (3) find independent risk factors for crude mortality in AP patients; and (4) describe the rate of antibiotic resistance inMDR-PA strains.

## Method

### Study design, setting, and ethics

At two tertiary referral centers, a retrospective analysis with a 1:2 case-control ratio in AP patients infected with MDR-PA was performed. The control group was age (± 2) and gender matched AP patients without MDR-PA infections. The clinical information we collected, included age, sex, etiology, classification of AP, referred patients, days from the onset of AP to admission, fungal infections, laboratory variables at admission, multiple organ failure (MOF), invasive mechanical ventilation, ICU stays, hospitalization, major complications, and mortality. The data collected were from Xiangya Hospital and Third Xiangya Hospital, which are both tertiary-care teaching hospitals, attached to the Central South University located in Changsha, China. The clinical information on AP patients with and without MDR-PA infections was collected from both hospitals from September 1st, 2017 to January 1st, 2022. The role of MDR-PA and carbapenem resistance among AP patients, risk factors of overall mortality, and distribution and drug-resistant rates of causative pathogens were investigated.

Drawing on previous studies and expert opinion, we estimated that approximately 0.1% of AP patients are suffered from *P. aeruginosa* infections. To achieve a significance level of 0.05 and a power of 80%, we calculated a minimum sample size of 69 patients would be required to detect a significant association between AP patients with and without *P. aeruginosa* infections in our study population.

The Institutional Review Board of Xiangya Hospital (No.202,103,047) and the Third Xiangya Hospital (No. 21,019) gave their approval prior to the commencement of the study. The need for written informed consent was eliminated for all patients due to the retrospective nature of the study.

### Definitions

The classification of AP, followed the Revised Atlanta Classification, which is defined as follows: (1) mild AP: the absence of either organ failure (OF) or local/systemic complications; (2) moderately severe AP: OF that resolves within 48 h and/or local or systemic complications without persistent OF, and (3) SAP: persistent OF > 48 h. The etiology criteria (used for the study) included the following: (1) hypertriglyceridemia: triglycerides more than 5.6 mmol/l without any other clear pathogeny; (2) gallstones: cholelithiasis or choledocholithiasis based on contrast-enhanced computerized tomography; and (3) alcoholism: regular drinking of alcohol (> 50 g/day) [5]. OF was defined for the 3 organ systems (respiratory, cardiovascular, and renal) according to the modified Marshall Score. MOF referred to failure occurring in more than one organ system [19]. The sites of infections were determined from consideration of inflammatory clinical characteristics and positive results from specimens. The primary infection sites were defined as the main infection sites [20]. The presence of infections was diagnosed based on the inflammatory clinical manifestations and positive results of specimens according to the criteria of the US Centers for Disease Control and Prevention. Drug resistance to either meropenem or imipenem was based on the definition of carbapenem resistance (minimum inhibitory concentration > 2 µg/ml). Infectious pancreatic necrosis was defined as the presence of gas bubbles within the (peri)pancreatic necrosis on computed tomography and a positive culture of (peri)pancreatic necrotic fluid obtained during the first intervention [20]. Isolations of MDR-PA were defined as positive specimens obtained from blood, bronchoalveolar fluid and the first drainage of the infectious pancreatic necrosis. Intermediate susceptibility to the antibiotics was considered as resistance [21, 22].

### Patients

Altogether, 23 patients with MDR-PA infections were classified in the case group and 46 patients without MDR-PA infections, into the control group. The interdisciplinary team evaluated and treated each AP patient upon admission in accordance with the latest international standards. In accordance with the Revised Atlanta Classification, AP was diagnosed and categorized via clinicians [5]. Fine-needle aspiration was never employed in either hospital to identify ‘suspect’ infected pancreatic necrosis in AP patients. Based on the detection and testing for drug resistance of MDR-PA strains, the antibiotic regimen of the initial therapy in 3–5 days, was documented as being promptly modified treatment based on the patient’s status [23]. Carbapenem (extended infusion and high-dose) was regarded as an appropriate choice for CRPA infections [24]. Identification and drug-resistant testing of MDR-PA strains were performed via the Vitek-2 system and microbroth dilution method, respectively [25].

### Statistical analysis

Categorical variables were characterized by using absolute numbers and percentages, and correlated using the χ2-test or Fisher’s exact test. Continuous variables were described with the standard deviation and categorical variables, and calculated using the Student’s t-test. To evaluate the correlation, the odds ratio (OR) and 95% confidence interval (CI) were performed. Statistical significance was defined as a p-value of 0.05. SPSS 24.0 was utilized to analyze the data (IBM SPSS Statistics, IBM Corp., Armonk, NY, US).

## Results

### Clinical characteristics and outcome of AP patients

In Table 1, the average age of AP patients without MDR-PA infections was 46.9 ± 10.9 years, compared to 51.9 ± 11.4 years for AP patients with MDR-PA infections. There were 47 (68.1%) males in our cohort, including 31 (67.4%) males in the group without MDR-PA infections and 16 (69.6%) males in the group with MDR-PA infections. The leading etiology was hypertriglyceridemia (n = 28, 40.6%), followed by gallstones (n = 24, 34.3%), alcoholism (n = 3, 4.3%), and others (n = 14, 20.3%). There were 34 (49.3%) moderately severe AP and 35 (50.7%) SAP patients in the study, including 22 of 46 (47.8%) SAP patients without MDR-PA infections and 13 of 23 (56.5%) SAP patients with MDR-PA infections. Fifty-four (78.3%) AP patients were referred from other hospitals. The days from the onset of AP to admission among AP patients with MDR-PA infections were all significantly longer than those of AP patients without MDR-PA infections (28.1 ± 4.9 days vs. 13.6 ± 17.9 days, *P* = 0.011). Fungal infections were more common in the AP patients with MDR-PA infections (n = 10, 43.5%) than without MDR-PA infections group (n = 2, 4.3%), respectively (*P* < 0.001). The difference in hematocrit between AP patients with (29.2 ± 8.1) and without MDR-PA infections (35.1 ± 8.2) was statically significant (*P* = 0.006). MOF occurred more frequently in the MDR-PA infections group with statistical significance (*P* = 0.039). Invasive mechanical ventilation in the with MDR-PA infections group was more frequent than without MDR-PA infections group (52.2% vs. 10.0%, *P* < 0.001). In comparison to the group without MDR-PA infections, the length of hospital stays (including ICU stays) was significantly longer in the MDR-PA infection group. The major complications in all these AP patients were hemorrhage (n = 8, 5.8%), intestinal leakage (n = 4, 5.8%), and pancreatic fistula (n = 3, 4.3%), respectively. Five patients (21.7%) in the group with MDR-PA infections and three patients (6.5%) in the group without MDR-PA infections experienced hemorrhage (*P* = 0.144). Both the group with MDR-PA infections and the group without MDR-PA infections had patients with intestinal leakage (3 (13.0%) vs. 1 (2.2%), respectively, *P* = 0.202), and the pancreatic fistula was experienced by two patients (8.7%) in the group with MDR-PA infections and one patient (2.2%) in the group without MDR-PA infections (*P* = 0.531). The overall mortality rate of AP patients was 15.9% in our study. The mortality of the patients with MDR-PA infections was significantly higher than those without MDR-PA infections (30.4% vs. 8.7%, *P* = 0.048).

**Table 1:** Clinical characteristics and comparison between with and without MDR-PA infections in AP patients

Characteristics	Total	Without MDR-PA infections (n = 46)	With MDR-PA infections (n = 23)	*P*
Age, years (mean ± SD)	48.6 ± 11.2	46.9 ± 10.9	51.9 ± 11.4	0.087
Sex, *n* (%)				0.855
Male	47 (68.1)	31 (67.4)	16 (69.6)	
Female	22 (31.9)	15 (32.6)	7 (30.4)	
Etiology, *n* (%)				0.561
Hypertriglyceridemia	28 (40.6)	20 (43.5)	8 (34.8)	
Gallstone	24 (34.8)	14 (30.4)	10 (43.5)	
Alcoholism	3 (4.3)	3 (6.5)	0	
Others	14 (20.3)	9 (19.6)	5 (21.7)	
Classification of AP, *n* (%)				0.496
Moderately severe AP	34 (49.3)	24 (52.2)	10 (43.5)	
SAP	35 (50.7)	22 (47.8)	13 (56.5)	
Referred patient, *n* (%)	54 (78.3)	34 (73.9)	20 (87.0)	0.216
Days from the onset AP to admission, days (mean ± SD)	18.4 ± 22.5	13.6 ± 17.9	28.1 ± 4.9	0.011*
Fungal infections, *n* (%)	12 (17.4)	2 (4.3)	10 (43.5)	< 0.001*
Laboratory variables at admission, (mean ± SD)				
Albumin, g/L	30.2 ± 4.9	30.9 ± 4.7	28.6 ± 4.9	0.051
Platelet, 10^3^/mm^3^	225.2 ± 117.8	228.9 ± 117.4	217.7 ± 120.8	0.715
Neutrophil, 10^3^/mm^3^	10.4 ± 6.6	10.3 ± 6.3	10.8 ± 7.3	0.765
Hematocrit, %	33.1 ± 8.6	35.1 ± 8.2	29.2 ± 8.1	0.006*
Blood urea nitrogen, mmol/L	8.4 ± 7.1	6.9 ± 4.6	11.1 ± 10.0	0.056
Lymphocyte, 10^3^/mm^3^	1.1 ± 0.8	1.2 ± 0.9	1.0 ± 0.6	0.244
MOF, *n* (%)	13 (18.8)	5 (10.9)	8 (34.8)	0.039*
Antibiotic treatments, *n* (%)				0.108
None	7 (10.1)	7 (15.2)	0	
Carbapenem	22 (31.9)	14 (30.4)	8 (34.8)	
Cephalosporins with beta-lactam	19 (27.5)	19 (41.3)	0	
Penicillin with beta-lactam	8 (11.6)	2 (4.3)	6 (26.1)	
Carbapenem + tigecycline	6 (8.7)	1 (2.2)	5 (21.7)	
Penicillin with beta-lactam + tigecycline	2 (2.9)	1 (2.2)	1 (4.3)	
Tigecycline	2 (2.9)	1 (2.2)	1 (4.3)	
Penicillin with beta-lactam + carbapenem	1 (1.4)	0	1 (4.3)	
Polymyxin	1 (1.4)	0	1 (4.3)	
Quinolone	1 (1.4)	1 (2.2)	0	
Invasive mechanical ventilation, *n* (%)	17 (24.6)	5 (10.9)	12 (52.2)	< 0.001*
ICU stays, days (mean ± SD)	10.2 ± 15.8	4.8 ± 8.5	20.9 ± 21.1	0.002*
Hospitalization, days (mean ± SD)	36.1 ± 22.8	29.9 ± 20.6	48.2 ± 22.6	0.001*
Intervention for pancreatic necrosis, *n* (%)				0.003*
Conservative therapy	9 (13.0)	8 (17.4)	1 (4.3)	
Only percutaneous catheter drainage	20 (29.0)	17 (37.0)	3 (13.0)	
Only endoscopic transluminal drainage	7 (10.1)	5 (10.2)	2 (8.7)	
Percutaneous catheter drainage step-up to minimal access retroperitoneal necrosectomy	18 (26.1)	6 (13.0)	12 (52.2)	
Percutaneous catheter drainage step-up to video-assisted retroperitoneal debridement	3 (4.3)	2 (4.3)	1 (4.3)	
Endoscopic transluminal drainage to endoscopic transluminal necrosectomy	5 (7.2)	5 (10.9)	0	
Open necrosectomy	3 (4.3)	3 (6.5)	0	
Step-up to open necrosectomy	4 (5.8)	0	4 (17.4)	
Major complications, *n* (%)				
Hemorrhage	8 (11.6)	3 (6.5)	5 (21.7)	0.144
Intestinal leakage	4 (5.8)	1 (2.2)	3 (13.0)	0.202
Pancreatic fistula	3 (4.3)	1 (2.2)	2 (8.7)	0.531
Mortality, *n* (%)	11 (15.9)	4 (8.7)	7 (30.4)	0.048*

**Abbreviations:** SD, standard deviation.

Note:^*^P values are statistically significant between with and without MDR-PA infections group.

### Comparison between CRPA and carbapenem-sensitive ***P. aeruginosa*** (CSPA) group

As shown in Table 2, compared to the CSPA group, the incidence rate of MOF was statistically higher in the CRPA group (57.1% vs. 0%, respectively, *P* = 0.018). The rate of prophylactic use of carbapenem for 3 days was remarkably higher in the CRPA group compared with the CSPA group (50.0% vs. 0%, respectively, *P* = 0.019). The ICU stays (26.3 ± 21.9 days vs. 12.7 ± 18.1 days, *P* = 0.120), and the hospital stays (54.4 ± 24.3 days vs. 38.6 ± 16.5 days, *P* = 0.076) in the CRPA group were longer than in the CSPA group without reaching a statistical difference. There was no significant difference in mortality between the CRPA group and the CSPA group (6 (42.9%) vs. 1 (11.1%), respectively, *P* = 0.250).

**Table 2:** Clinical characteristics and comparison between CRPA and CSPA infections among 23 MDR-PA infected AP patients

Characteristics	CSPA (n = 9)	CRPA (n = 14)	*P*
Age, years (mean ± SD)	49.1 ± 11.9	53.7 ± 11.1	0.365
Sex, *n* (%)			0.250
Male	8 (88.9)	8 (57.1)	
Female	1 (11.1)	6 (42.9)	
Etiology, *n* (%)			0.512
Hypertriglyceridemia	3 (33.3)	5 (35.7)	
Gallstone	5 (55.6)	5 (35.7)	
Others	1 (11.1)	4 (28.6)	
Classification of AP, *n* (%)			0.613
Moderately severe AP	5 (55.6)	5 (35.7)	
SAP	4 (44.4)	9 (64.3)	
Referred patient, *n* (%)	8 (88.9)	12 (85.7)	0.998
Fungal infection, *n* (%)	2 (22.2)	8 (57.1)	0.223
MOF, *n* (%)	0	8 (57.1)	0.018*
Primary site of infections, *n* (%)			0.265
Lung	4 (44.4)	9 (64.3)	
Pancreas (peri)	5 (55.6)	4 (28.6)	
Bloodstream	0	1 (7.1)	
Prophylactic antibiotics, *n* (%)			0.019*
Wide-spectrum antibiotics for 3 days	9 (100.0)	7 (50.0)	
Carbapenem for 3 days	0	7 (50.0)	
Intervention for pancreatic necrosis, *n* (%)			0.745
Conservative therapy	1 (11.1)	0	
Only percutaneous catheter drainage	1 (11.1)	2 (14.3)	
Only endoscopic transluminal drainage	1 (11.1)	1 (7.1)	
Percutaneous catheter drainage step-up to minimal access retroperitoneal necrosectomy	5 (55.6)	7 (50.0)	
Percutaneous catheter drainage step-up to video-assisted retroperitoneal debridement	0	1 (7.1)	
Step-up to open necrosectomy	1 (11.1)	3 (21.4)	
ICU stays, days (mean ± SD)	12.7 ± 18.1	26.3 ± 21.9	0.120
Hospitalization, days (mean ± SD)	38.6 ± 16.5	54.4 ± 24.3	0.076
Major complications, *n* (%)			
Hemorrhage	0	5 (35.7)	0.116
Intestinal leakage	1 (11.2)	2 (14.3)	1.000
Pancreatic fistula	0	2 (14.3)	0.668
Mortality, *n* (%)	1 (11.1)	6 (42.9)	0.250

Note:^*^P values are statistically significant.

### Risk factors for mortality

In Table 3, risk factors for mortality in AP patients, was elucidated using which were shown by the univariate analysis, included the severe category of AP (*P* = 0.017) and MDR-PA infections (*P* = 0.028). Both of them were statistically major risk factors were identified by the multivariate analysis (OR = 13.624; 95% CIs = 1.567-118.491, *P* = 0.018; OR = 4.788; 95% CIs = 1.107–20.709, *P* = 0.036).

**Table 3:** Univariate and multivariate analysis of risk factors for mortality in AP patients

Variable	Survival (*n* = 61)	Mortality (*n* = 11)	Univariate analysis			Multivariate analysis	
			OR (95% CIs)	*P*		OR (95% CIs)	*P*
Age ≥ 60 years old, *n* (%)	7 (12.1)	3 (27.3)	2.732 (0.583–12.799)	0.202			
Male, *n* (%)	39 (67.2)	8 (72.7)	1.299 (0.309–5.460)	0.721			
Referral from other hospitals, *n* (%)	45 (77.6)	9 (81.8)	1.300 (0.249–6.781)	0.756			
Severe category of AP, *n* (%)	25 (43.1)	10 (90.9)	13.200 (1.584–110.004)	0.017*		13.624 (1.567–118.491)	0.018*
Fungal infections, *n* (%)	8 (13.8)	4 (36.4)	3.571 (0.848–15.035)	0.083			
MDR-PA infections, *n* (%)	16 (27.6)	7 (63.6)	4.594 (1.183–17.840)	0.028*		4.788 (1.107–20.709)	0.036*

**Abbreviations:** OR, odds ratio; CI, confidence interval.

**Note:** *P values are statistically significant.

### Distribution and drug-resistant rates of MDR-PA strains

In Table 4, the pancreas (n = 14) and lung (n = 12) were the common sites of infection. MDR-PA strains were highly resistant to 7 of the 10 antibiotics (more than 20%), however, the resistance rates were low in amikacin (7.4%), tobramycin (3.7%), and gentamicin (18.5%). The resistance rates of MDR-PA strains to imipenem and meropenem were more than 50%.

**Table 4:** Resistance rates of 27 strains to 10 antibiotics according to the different sites of infections among MDR-PA infected AP patients

Antimicrobial	Pancreas (*n* = 14)	Lung (*n* = 12)	Bloodstream (*n* = 1)	Total strains (*n* = 27)
Amikacin	2 (14.3)	0	0	2 (7.4)
Aztreonam	10 (71.4)	12 (100)	1 (100)	23 (85.2)
Ciprofloxacin	4 (28.6)	7 (58.3)	0	11 (40.7)
Gentamicin	3 (21.4)	2 (16.7)	0	5 (18.5)
Imipenem	5 (35.7)	8 (66.7)	1 (100)	14 (51.9)
Meropenem	7 (50.0)	8 (66.7)	0	15 (55.6)
Levofloxacin	4 (28.6)	6 (50.0)	0	10 (37.0)
Tobramycin	1 (7.1)	0	0	1 (3.7)
Piperacillin-tazobactam	4 (42.9)	10 (83.3)	0	16 (59.3)
Macrodantin	14 (100)	12 (100)	1 (100)	27 (100)

**Note:** Values are no. (%) of resistant strains, except as indicated.

## Discussion

*P. aeruginosa*, especially MDR-PA, is classified as the most lethal causative non-lactose-fermenting Gram-negative bacilli which is capable of acquiring resistance to multiple categories of antibiotics [26]. In the past decade, MDR-PA represented a frequent and challenging nosocomial pathogen with consistently high mortality rates worldwide [26]. In our study, we showed that MDR-PA infections had a crude mortality rate up to 30.4%, and the crude mortality rate reached 42.9% when MDR-PA infections developed CRPA infection. The possible reasons for our findings of high mortality might have been due to the capacity of strains to acquire resistance to common antibiotics and the striking virulence through myriads of mechanisms [27].

Similar to Datta et al., we found that the days from onset to admission, length of hospital and ICU stays of AP patients with MDR-PA infections were significantly longer than those without MDR-PA infections, which might result in an increased risk of nosocomial infections [28]. On the other hand, MDR-PA infections can increase the difficulty of treatment, thus prolonging the length of hospitalization and/or ICU stays, which could suggest a mutual cause-and-effect relationship with MDR-PA infections [29]. We also found that the incidences of MOF in MDR-PA infections were higher than those in the group without MDR-PA infections. According to a previous study, MOF, particularly respiratory failure, needed invasive treatment, such as mechanical ventilation. This resulted in an increased *P. aeruginosa* colonization due to mucosal barrier breakdown, which therefore increased the risk of respiratory tract infections in the MDR-PA group [22]. Similar to Shi et al., it had been proven that MOF strongly correlated with the severity of AP and played a key role in the prognosis of AP patients [30]. This could possibly be due to following reasons: (1) SAP patients might suffer from invasive operations and longer ICU stays with the heavy use of antibiotics, which subsequently enhanced the risk of MDR-PA infections due to their immunosuppressed state, and (2) severe infections could have resulted in organ failure which developed moderately severe AP into SAP. Unsurprisingly, the multivariate analysis in our study revealed that both MDR-PA infections and the severity of AP were independent risk factors for overall mortality. The severity of AP outperformed MDR-PA infections with higher OR as the stronger risk factor of mortality. This thus suggests more attention is needed on this in future work.

In line with a previous study, we also found that MOF and the prescription of carbapenem antibiotics were significantly related to the CRPA infections in AP patients with MDR-PA infections [20]. This should alert the clinician to focus the MOF, as a clinical marker for antibiotics’ prescription to suspected infections, and enhance the administration of carbapenem antibiotics as a prevention for the drug-resistance progression [22, 31–33]. In addition, the American Gastroenterological Association guideline shows that the prophylactic use of antibiotics cannot reduce the complications and mortality of AP patients [34]. ‘Survival of the fittest’ was a consequence of an immense genetic plasticity of MDR-PA pathogens that triggered specific responses that resulted in mutational adaptations, acquisition of genetic material, or alteration of gene expression producing resistance to virtually all antibiotics currently available [35]. MDR-PA sharing the environment with these molecules harbored intrinsic genetic determinants of resistance, and there was robust evidence suggesting that such “environmental resistome” was a prolific source of the acquisition of drug-resistance genes in clinically relevant MDR-PA which in turn, is reflected in a higher bacterial minimum inhibitory concentration [35]. Furthermore, this genetic exchange has been implicated in the dissemination of resistance to many frequently antibiotics’ prescription, and overuse of antibiotics for AP patients in China which ranked 1st in the world might be the main cause of reason for MDR-PA infections in our study [36]. The appropriate use of antibiotics to treat confirmed infections can decrease the risk of sepsis, organ failure, and mortality in AP patients [37].

In accordance with a previous study of other MDR bacterial infections among AP patients, we showed that the pancreas and lung were the most common sites of MDR-PA infections [20]. This indicates a similar distribution of MDR-PA with other Gram-negative bacterial and enhances the importance of strengthening the management of both pancreatic and extra-pancreatic infections in the course of AP [7, 24, 38]. Consistent with other studies, we also found that the resistance rate of MDR-PA strains to amikacin, tobramycin, and gentamicin was lower than that of other drugs (< 20%) [13, 39–41]. However, a recent study did highlight that the drug resistance rate of MDR-PA to amikacin and gentamicin had an upward trend, which might alert clinicians to enhance the administration of the both antibiotics [42]. In addition, the nephrotoxicity of amikacin should be considered. It may aggravate renal insufficiency in AP patients, and is associated with higher mortality in AP patients [43, 44]. Prior to the results of bacterial culture and drug sensitivity testing being obtained, the patients with suspected infections were at high risk of MDR-PA infections, including invasive mechanical ventilation combined with organ failure. It would be recommended to treat these patients with the above 3 antibiotics as an empirical antimicrobial regimen. Moreover, organ function should also be considered as an important factor for the appropriate dose. Antibiotics should only be prescribed when a comprehensive evaluation of all available information. This included clinical manifestations, physical examination, laboratory parameters and imaging findings, which strongly suggested a secondary infection in AP patients. They though should not be overused due to the risk of increasing antimicrobial resistance.

To the best of our knowledge, our study is the first to investigate the impact of MDR-PA infections on AP Patients. Nevertheless there are potential limitations within the study. Firstly, due to the retrospective case-control nature of the study, there could be bias and deficient variables. We didn’t include some factors influencing the mortality of AP, such as intra-abdominal pressure and abdominal compartment syndrome. Our case-control study was matched with age and gender without APACHEII scores which might induce some selection bias due to its nature. We used the latest precise definitions as stated in the methods, which would decrease some of the bias. Secondly, the limited sample size of the case group gave a wide range of CIs which may reduce the statistical power. We performed a sample size estimation to ensure the size was sufficient. However, we still didn’t have a sufficient number of AP patients with sensitive *P. aeruginosa* infections for further comparative analysis due to both the limited number of patients and inherent resistance of *P. aeruginosa* strains. Thirdly, in the era of MDR bacterial infections, novel molecular technology should be performed to promote rapid diagnoses of infections and reveal the antibiotic resistance mechanisms of MDR-PA strains. Fourthly, the timing of intervention for infected pancreatic necrosis was lacking, due to the fact that the clinicians chose a conservative treatment for pancreatic necrosis. Finally, our findings should be carefully interpreted in the medical centers with a low incidence rate of MDR-PA infections due to our limited sample size.

## Conclusions

In AP patients, severe categories of AP and MDR-PA infections were both independent risk factors for mortality. Inappropriate use of carbapenem antibiotics and MOF were related to CRPA infections. Amikacin, tobramycin, and gentamicin are recommended for treatment of AP patients with MDR-PA infections.

## Data Availability

The datasets used and/or analyzed during the current study are available from the corresponding author on reasonable request.

## Abbreviations

AP: Acute pancreatitis
MDR-PA: multi-drug resistant
MDR-PA: Multi-drug resistant *Pseudomonas aeruginosa*
MOF: Multiple organ failure
SAP: Severe acute pancreatitis
ICU: Intensive care unit
CRPA: Carbapenem-resistant *Pseudomonas aeruginosa*
OF: Organ failure
OR: The odds ratio
CI: Confidence interval
CSPA: Carbapenem-sensitive P. aeruginosa
